# Distinct functional roles of *Lactobacillus* strains in modulating lipid metabolism, adipose accumulation, inflammatory responses, and carcass characteristics in heavy finishing pigs

**DOI:** 10.1016/j.aninu.2026.03.006

**Published:** 2026-07-31

**Authors:** Boyang Wan, Qiuyu Zeng, Linjuan He, Xu Qin, Mingyang Tan, Enfa Yan, Jialong Liao, Zhongchao Li, Xin Zhang, Jingdong Yin

**Affiliations:** a State Key Laboratory of Animal Nutrition and Feeding, College of Animal Science and Technology, China Agricultural University, Beijing 100193, China; b Guizhou Fuzhiyuan Company, Guiyang 550200, Guizhou, China; c Molecular Design Breeding Frontier Science Center of the Ministry of Education (MOE), Beijing 100193, China

**Keywords:** *Lactobacillus reuteri*, *Lactobacillus johnsonii*, Lipid metabolism, Subcutaneous fat accumulation, Carcass characteristics, Pig

## Abstract

Supplementation with *Lactobacillus* strains attracts intense interest for its potential in regulating fat accumulation in both humans and animals. However, the functional disparities among *Lactobacillus* strains remain poorly understood. This study examined the impact of dietary supplementation with *Lactobacillus reuteri* (*L. reuteri*) and *Lactobacillus johnsonii* (*L. johnsonii*) on lipid metabolism, carcass traits, and meat quality in heavy finishing pigs. A total of 288 finishing pigs (equal numbers of castrated barrows and gilts; initial body weight = 109.88 ± 1.58 kg) were randomly allocated to three dietary treatments with six replicate pens and 16 pigs per pen. Pigs were fed a basal diet (CON), or the basal diet supplemented with *L. reuteri* or *L. johnsonii* at a daily dose of 5 × 10^10^ CFU per pig, respectively, over a 42-d experimental period. The experimental results demonstrated that compared with CON, both probiotic strains significantly decreased serum levels of cortisol and pro-inflammatory factors including tumor necrosis factor‑α (TNF-α), interleukin‑1β (IL-1β) and interleukin‑17 (IL-17), while significantly elevating the levels of anti-inflammatory factor interleukin‑4 (IL-4) and total antioxidant capacity (T-AOC) (*P* < 0.05), and reduced the concentration of saturated fatty acids in longissimus thoracis (LT) (*P* = 0.014). *L. johnsonii* supplementation significantly reduced backfat thickness at the 10th-rib (*P* = 0.040) and last-rib backfat (*P* = 0.025), significantly increased fat-free lean index (*P* = 0.015), and significantly decreased adipocyte size in subcutaneous adipose tissue (*P* < 0.001), accompanied by the activation of the AMPK/PPARγ signaling pathway. Compared with CON, *L. reuteri* supplementation significantly reduced the shear force (*P* = 0.043) and intramuscular fat (IMF) content (*P* = 0.026) of LT muscle of finishing pigs and significantly increased free valine, methionine, and isoleucine concentrations in fresh meat (*P* < 0.05). In conclusion, the two strains of *Lactobacillus* exhibited distinct characteristics. Specifically, *L. johnsonii* primarily promoted lipid catabolism while simultaneously suppressing lipid anabolism by activating the AMPK/PPARγ signaling pathway, leading to a reduction in backfat thickness. In contrast, *L. reuteri* exhibited stronger focus on improving meat quality traits, such as tenderness and amino acid composition. This study offers a valuable insight into the specific application of probiotics in high-quality pork production.

## 1 Introduction

Pork constitutes the predominant choice of meat consumption across the majority of the global regions, attributed to its superior nutritional properties and palatability (Elmadfa and Meyer, 2017). Genetic selection and systematic crossbreeding programs have substantially decreased backfat thickness while increasing lean percentage in modern lean-type pigs (Gozalo-Marcilla et al., 2021). Nowadays, the average slaughter weight of pigs has consistently increased, rising from 110 kg to over 130 kg or even reaching 140 to 150 kg in some regions. In this case, maintaining optimal backfat thickness and leanness remains crucial for producing high-grade pig carcasses (Wang et al., 2023b).

Subcutaneous fat deposition exhibits a significant negative correlation with both lean percentage and pork production efficiency in pigs, and excessive accumulation leads to a decline in carcass quality grading (Hoa et al., 2021). Consistently maintaining optimal backfat thickness and lean percentage remains a major challenge, especially in the production of heavy pigs. Dietary nutritional supply serves as a critical factor influencing body composition, backfat accumulation, and meat quality in finishing pigs (Yan et al., 2023). The application of functional nutritional substitutes, including various probiotics in the pig industry, has received increasing attention (Minj et al., 2021).

Previous studies on *Lactobacilli* in pigs mainly focused on improving intestinal health and facilitating growth in weaned piglets (Dowarah et al., 2017; Saha et al., 2024). The specific taxa in porcine cecal microbial community exhibited a significant correlation with backfat thickness and abdominal fat accumulation (He et al., 2016). It has been reported that dietary supplementation with *Lactobacilli* improved the functional profile of intestinal microbiota, enhanced the edible quality of meat, including color score, marbling score, and redness (a∗) value in finishing pigs (Meng et al., 2010), as well as pork flavor (Liu et al., 2023). Researches in mice and humans demonstrated that *Lactobacillus* strains and their metabolites could modulate fat deposition by regulating lipid metabolism (He et al., 2024; Liu et al., 2022; Zhong et al., 2022). However, how to apply *Lactobacillus* strain to modulate body composition and fat deposition remains to be elucidated. It was hypothesized that *Lactobacillus* strains supplementation could regulate lipid metabolism in heavy pigs, thereby reducing subcutaneous fat accumulation and improving carcass characteristics. Accordingly, the regulatory effects of dietary supplementations with *Lactobacillus reuteri* (*L. reuteri*) and *Lactobacillus johnsonii* (*L. johnsonii*) on subcutaneous backfat deposition in heavy pigs were examined, and the underlying mechanisms were elucidated. The study offered valuable insights into the regulatory role of *Lactobacillus* probiotics in lipid metabolism and fat accumulation, with potential applications in controlling subcutaneous backfat deposition and enhancing carcass traits and meat quality in heavy pigs.

## 2 Materials and methods

### 2.1 Animal ethics statement

The experiment was carried out in strict compliance with the Chinese Guidelines for Animal Welfare and Experimental Protocols, and the experimental protocol was approved by the Animal Care and Use Committee of China Agricultural University in 2015 (approval ID: AW42015202-1-01).

### 2.2 Animals and diets

A total of 288 crossbred finishing pigs (Duroc × Landrace × Large White pigs), with initial body weight (BW) of 109.88 ± 1.58 kg, were randomly assigned to three dietary treatments, with six replicates (pens) per treatment, consisting of 16 pigs each pen (1:1 barrows and gilts), receiving the basal diet as the control group (CON), the basal diet supplemented with freeze-dried *L. reuteri* powder (1 × 10^10^ CFU/g freeze-dried powder, 5 × 10^10^ CFU/d per pig), and the basal diet with freeze-dried *L. johnsonii* (1 × 10^10^ CFU/g freeze-dried powder, 5 × 10^10^ CFU/d per pig). The probiotics *L. reuteri* and *L. johnsonii* were purchased from Zhongke Jiayi Biological Engineering Co., Ltd. (Weifang, Shandong, China). All experimental diets were meticulously formulated to meet or surpass the nutrient requirements for swine as established by the NRC (2012) (Table 1, Table 2). Dry matter content of basal diets was determined following method 934.01 (AOAC, 2006). Crude protein was analyzed using a Kjeldahl nitrogen determination system (Kjeldahl 8100, FOSS Analytical A/S, Hillerød, Denmark) according to method 984.13 (AOAC, 2006). Amino acid contents in diets were analyzed by high-performance liquid chromatography (HPLC; L-8900, Hitachi High-Tech Corp., Tokyo, Japan) as described by Heinrikson and Meredith (1984). Calcium, total phosphorus, standardized total tract digestibility of phosphorus, and net energy levels were calculated according to NRC (2012). As for probiotics supplementation, *L. reuteri* and *L. johnsonii* were freshly dissolved in distilled water to prepare a *Lactobacillus* solution containing 8 × 10^8^ CFU/mL. This solution was evenly sprayed onto the surface of the 1/10 feeding amount of pelletized feed, and then manually mixed with the remaining feed to produce a feed containing with 2.5 × 10^7^ CFU *Lactobacillus*/g diet. During the first two weeks of the trial, probiotics were supplemented to the pigs daily in the morning. Thereafter, they were supplemented every three days in the morning until the end of the trial. Throughout the 42-d trial, pigs were permitted to ad libitum access to feed and water. The initial and final BW of all pigs, and feed intake per pen were meticulously recorded to calculate the average daily gain (ADG), average daily feed intake (ADFI), and feed:gain ratio (F:G).

**Table 1:** Ingredients and nutrient levels of diets (%, as-fed basis).

Ingredients	Content	Nutrients	Content
Corn	54.55	**Calculated nutrients**^2^	
Soybean meal	6.34	Net energy, MJ/kg	10.67
Wheat flour	15.00	Organic matter	84.11
Distillers dried grain	3.00	Calcium	0.59
Rice bran	12.40	Total phosphorus	0.57
Rapeseed meal	4.00	STTD phosphorus	0.29
Soybean oil	0.50	**Analyzed nutrients**	
L-Lysine HCl	0.80	Dry matter	88.32
L-Threonine	0.18	Crude protein	13.59
L-Tryptophan	0.05	Lysine	0.92
DL-Methionine	0.06	Methionine + cysteine	0.49
Valine	0.02	Threonine	0.59
Limestone	0.90	Tryptophan	0.16
Dicalcium phosphate	0.75	Valine	0.62
Slat	0.45	Isoleucine	0.46
Premix^1^	1.00	Arginine	0.67
Total	100.00	Histidine	0.30
		Leucine	1.02
		Phenylalanine	0.52

STTD = standardized total tract digestibility.

1 Premix provided these amounts of vitamins and minerals per kg on an as-fed basis for finishing pigs: vitamin A, 6450 IU; vitamin D_3_, 2250 IU; vitamin E, 18 IU; vitamin K_3_, 1.5 mg; vitamin B_1_, 1.65 mg; vitamin B_2_, 6 mg; vitamin B_6_, 3 mg; vitamin B_12_, 0.12 mg; biotin, 0.16 mg; folic acid, 1.95 mg; pantothenic acid, 13.95 mg; nicotinic, 19.5 mg; Cu (CuCl_2_), 12.51 mg; Fe (FeSO_4_ H_2_O), 105 mg; Mn (MnSO_4_·H_2_O), 41.48 mg; Zn (ZnSO_4_ H_2_O), 61.66 mg; I (Ca(IO_3_)_2_), 0.33 mg; Se (Na_2_SeO_3_), 0.38 mg.

2 The values were calculated using data provided by NRC (2012).

**Table 2:** Fatty acid profile of diets (mg/g feed).

Ingredients	Content	Ingredients	Content
C8:0	0.03	C21:0	0.05
C10:0	0.02	C20:3n6	0.01
C12:0	0.02	C20:4n6	0.04
C14:0	0.27	C20:3n3	0.01
C15:0	0.05	C22:0	0.11
C16:0	7.21	C20:5n3	0.26
C16:1	0.36	C22:1n9	0.09
C17:0	0.07	C23:0	0.03
C18:0	0.98	C24:0	0.16
C18:1n9c	11.05	C24:1	0.02
C18:2n6c	20.34	SFA	9.18
C18:3n3	0.98	MUFA	11.78
C20:0	0.18	PUFA	21.67
C20:1	0.26		

SFA = saturated fatty acid; MUFA = monounsaturated fatty acid; PUFA = polyunsaturated fatty acid.

### 2.3 Sample collection and preparation

After 42 d of feeding, one barrow with BW approximating the treatment mean was selected from each of the six replicate pens per dietary treatment, and fasted for 12 h. Venous blood was aseptically collected from the jugular veins of porcine subjects into sterile vacutainer tubes, followed by centrifugation at 3000 × *g* for 15 min at 4 °C to isolate the serum fraction. The serum samples were meticulously preserved at −20 °C for subsequent analysis.

Eight barrows per dietary treatment with BW close to the treatment mean were selected and transported to the local commercial abattoir (about 1.5 h of transportation). Due to the nearly fixed transportation schedules and limited resting space available for pigs in most Chinese abattoirs, a 4-h period is the longest feasible resting time. After resting for at least 4 h, pigs were scarified and then processed according to standard commercial slaughter protocols, including humane stunning, exsanguination, and evisceration.

Within 45 min post–slaughter, the longissimus thoracis (LT) muscle and the subcutaneous adipose tissue (SAT) from the right-side of each carcass between the 10th and 12th ribs were collected. Specifically, a portion (about 100 g) of the muscle was rapidly frozen at −20 °C for subsequent amino acid composition and fatty acid analysis, while a smaller portion (about 5 g) was preserved at −80 °C for quantitative real-time PCR (qRT-PCR), Western blot, and chemical analysis. Additionally, about 5 g of SAT samples were immediately fixed in Bouin’s solution, followed by paraffin embedding for subsequent hematoxylin and eosin (H&E) staining.

### 2.4 Carcass traits

The hot carcass weight, backfat thickness and loin eye area were measured on the slaughter line. The carcasses were then conveyed in refrigeration at 4 °C. The dressing percentage was determined as the ratio of hot carcass weight to slaughter weight of pigs. Backfat thickness was quantified at the thickest points of the shoulder, last rib, 6–7th ribs, 10th rib, and the last lumbar vertebra. Additionally, loin eye dimensions were recorded at the 10th rib, including both height and width. The loin eye area was subsequently measured with the formula:

$$\text{Loin}\;\text{eye}\;\text{area}\;\left({\text{cm}}^{2}\right)=\text{Loin}\;\text{eye}\;\text{height}\;\left(\text{cm}\right)\times \text{Width}\;\left(\text{cm}\right)\times 0.7\text{.}$$

The fat-free lean index was calculated with the following equation (National Pork Producers Council, 1994):

Fat-freeleanindex=50.767+[0.035×Hotcarcassweight(lb)]–[8.979×Lastribfatthickness(in.)].

### 2.5 Meat quality

Fresh meat characteristics were assessed through visual and instrumental methods following standardized commercial protocols. Meat color and marbling were scored at 24 h postmortem, with the color scale ranging from 1.0 (very pale, white) to 6.0 (dark purplish red) (National Pork Producers Council, 1999). Postmortem pH measurements included initial pH_45 min_ values, recorded using a portable pH meter (Testo 205, Testo SE & Co. KGaA, Lenzkirch, Germany) at 45 min, and ultimate pH_24 h_ values measured in a 4 °C chilled room after 24 h. Each sample was measured in triplicate, with the mean subsequently derived from the replicates. The pH meter was calibrated with standard buffers (pH 4.00 and 7.00) prior to sample measurements. Automatic temperature compensation was employed using the pH meter, thereby correcting temperature deviations. Instrumental color analysis was conducted at 45 min or 24 h postmortem. After undergoing a 30-min blooming process, the meat color was evaluated using a colorimeter (CR-410, Konica Minolta Holdings Inc., Osaka, Japan), equipped with an 8-mm measuring aperture and illuminated by a standardized xenon lamp. The instrument was set up with a D65 light source and a 2° standard observer to determine lightness (L∗), a∗, and yellowness (b∗) values according to the CIE Lab system (CIE, 1931). Before measurements, the instrument was calibrated using a white tile following the manufacturer’s specifications.

Longissimus thoracis muscle samples were excised from the right side of each carcass within 45 min post–slaughter, specifically between the 10th and 12th ribs to facilitate measurements of pork drip loss, cooking loss, and shear fore. Drip loss was determined as the percentage of weight difference before and after storage (4 °C, 24 h) according to Honikel (1998) as follows:

Driploss(%)=[(Initialweight-Finalweight)/Initialweight]×100.

For the evaluation of cooking loss, approximately 100 g of muscle samples were heated in a 70 °C water bath in the same batch for 30 min. After cooling to room temperature, samples were reweighed to calculate the percentage of weight loss. Subsequently, meat tenderness was evaluated using a digital tenderness meter (C-LM3B, Tenovo Instrument Equipment Co., Ltd., Beijing, China), with ten measurements taken per sample (1.27 cm^3^) to ensure accuracy. Approximately 50 g of LT muscle sample was thinly sliced (2–3 mm thick), weighed in aluminum boxes, and freeze-dried for 72 h using a vacuum lyophilizer (Freezone 4.5TM, Labconco Corp., Kansas, MO, USA). The lyophilized meat samples were subsequently ground into fine powder to facilitate subsequent analysis of intramuscular fat (IMF) content, as well as amino acid and fatty acid profiles in the pork. Intramuscular fat content was determined through Soxhlet extraction using petroleum ether (Extraction System B‑811, BÜCHI Labortechnik AG, Flawil, St. Gallen, Switzerland) according to previously described (Zhang et al., 2016), with results expressed as a percentage of fresh muscle weight.

### 2.6 Amino acid and fatty acid composition analysis

The content of free amino acids in LT muscle was analyzed using ultra-high-performance liquid chromatograph (ACQUITY UPLC I-Class/Xevo TQD IVD System, Waters Corporation, Milford, MA, USA) and high-resolution mass spectrometer (Q-Exactive Plus Hybrid Quadrupole Orbitrap, Thermo Fisher Scientific Inc., Waltham, MA, USA) as previously described (Xu et al., 2022). For extraction, approximately 300 mg of lyophilized meat was homogenized in a methanol-water solution (8:2, v/v) using six cycles of alternating ultrasonication (5 min) and room temperature incubation (1 min). After 2 h of ice incubation, samples were centrifuged (10,000 × *g*, 4 °C, 10 min) to obtain the supernatant for analysis. Hydrolyzed amino acid concentration in LT muscle was analyzed following the standard methodologies established by AOAC (2008).

About 150 mg of lyophilized meat or feed sample was subjected to fatty acid analysis by gas chromatography in accordance with AOAC (2008). Meat was subjected to extraction using 4 mL of an acetyl chloride-methanol solution (1:10, v/v), followed by the addition of 1 mL n-hexane and 1 mL internal standard fatty acid solution (1 mg/mL undecanoic, C11:0). The mixture was then incubated at 75 °C for a duration of 2 h. After reaction, 5 mL aliquot of a 70 g/L potassium carbonate solution was introduced, followed by centrifugation at 800 × *g* for 5 min. The supernatant was then analyzed using a gas chromatograph (HP 6890, Agilent Technologies Inc., Santa Clara, CA, USA) equipped with a DB-23 capillary column (100 m × 0.25 mm, 0.25 μm film thickness; Agilent Technologies Inc., Santa Clara, CA, USA). The analytical results were quantified as mg of fatty acids per 100 g muscle tissue.

### 2.7 Serum and subcutaneous fat biochemical parameters

The contents of triglyceride (TG), total cholesterol (TC), high-density lipoprotein (HDL), and low-density lipoprotein (LDL), glucose (GLU), total protein (TP), and albumin (ALB) in the serum were quantified using an automatic biochemistry analyzer (Beckman BS-420, Beckman Coulter Inc., Brea, CA, USA). The globulin (GLB) content was derived by subtracting the ALB value from the TP concentration.

In this study, all commercial kits used in the following analysis of serum and subcutaneous fat biochemical parameters, unless otherwise specified, were purchased from Nanjing Angle Gene Bioengineering Company (Nanjing, Jiangsu, China). The serum levels of insulin-like growth factor-1 (IGF-1; E31112P), and cortisol (E31032P), lipopolysaccharide (LPS; E31075P), tumor necrosis factor-α (TNF-α; E31006P), interleukin-1β (Il-1β; E31272P), interleukin-6 (IL-6; E31023P), interleukin-17 (IL-17; E31091P), interleukin-4 (Il-4; E31025P), and interleukin-10 (IL-10, E31026P) were determined according to the manufacturer’s guidelines. The levels of total antioxidant capacity (T-AOC; YH1246), superoxide dismutase (SOD; YH1202), catalase (CAT; YH1208), glutathione peroxidase (GSH-Px; YH1267), and the content of malondialdehyde (MDA; YH1217) in serum were determined using commercial kits. The enzyme activity of adipose triglyceride lipase (ATGL; E3298G), hormone-sensitive triglyceride lipase (HSL; E123509J), stearoyl-CoA desaturase 1 (SCD1; E301143S) and acetyl-CoA carboxylase (ACC; ZS3178) in the SAT of finishing pigs was measured using commercial kits according to the manufacturer’s instructions.

### 2.8 Histological examination

Sections approximately 5 μm thick were obtained from the SAT paraffin block using a Leica microtome (RM 2235, Leica Biosystems Nussloch GmbH, Nussloch, Germany) and precisely mounted onto poly-L-lysine-coated glass slides. The SAT tissue sections were subsequently processed through a standardized sequential dewaxing and rehydration protocol, utilizing a graded xylene series followed by a descending ethanol gradient (100%, 95%, 85%, and 75%). Following this, the sections were immersed in hematoxylin solution for 2 min, then rinsed extensively with running water until no residual stain was visible. To remove excess hematoxylin staining in the cytoplasm, the sections were subjected to differentiation using hydrochloric acid alcohol and followed by thorough rinsing in distilled water until the characteristic blue nuclear staining appeared. The sections were subsequently counterstained with eosin for 5 min. After dehydration, the tissue sections were meticulously sealed with a transparent resin and prepared for detailed observation and high-resolution imaging using a microscope (CK40, Olympus Corporation, Tokyo, Japan). Adipocyte areas were manually measured in ImageJ software (National Institutes of Health, Bethesda, MD, USA) (Schneider et al., 2012), with a minimum of 100 adipocytes analyzed per animal. To ensure accurate representation of mature adipocytes, objects with an area below 350 μm^2^ were excluded, as these likely consisted of stromal vascular cells or small adipocyte precursors. Adipocyte size frequency distributions were generated following established methods (Parlee et al., 2014). Size bins were defined in 1000 μm^2^ increments, and the frequency of adipocytes within each bin was calculated using Excel’s FREQUENCY function. The raw frequency counts were then transformed into percentages by computing the proportion of each bin count relative to the total number of adipocytes analyzed.

### 2.9 RNA extraction and qRT-PCR

Total RNA was isolated from LT muscle with a kit of RNAiso Plus (9109, TaKaRa Bio Inc., Dalian, Liaoning, China), followed by reverse transcription into cDNA employing the PrimeScript RT reagent kit (RR047A, TaKaRa Bio Inc., Dalian, Liaoning, China) through sequential incubations at 37 °C for 15 min and 85 °C for 5 s. SYBR Green-based qPCR was conducted using a qTOWER 2.2 real-time thermal cycler (Analytik Jena GmbH + Co., KG, Jena, Thuringia, Germany). Reaction mix tures (10 μL total volume) comprised 4 μL cDNA template, 0.25 μL forward and reverse primers each, 5 μL SYBR Premix Ex Taq II (Tli RNaseH Plus, RR820A, TaKaRa Bio Inc., Dalian, Liaoning, China), and 0.5 μL nuclease-free water. Thermocycling conditions included an initial denaturation phase (95 °C for 5 min) preceding 40 cycles of amplification (95 °C for 15 s, 60 °C for 15 s, 72 °C for 15 per cycle). The primer sequences for qRT-PCR were presented in Table S1, and *GAPDH* served as an internal reference gene. The full names of the abbreviations of all genes can be found in supplementary file abbreviations and full names of genes and proteins. The relative expression levels of the target genes were calculated by the 2^-△△Ct^ method (Schmittgen and Livak, 2008).

### 2.10 Western blot analysis

Subcutaneous adipose tissue tissues lysates were acquired by using radio immunoprecipitation assay lysis buffer with protease inhibitor mixture cocktail (HX1862–2, Huaxingbio Gene Technology Co., Ltd., Beijing, China). Protein concentration of SAT tissues lysates was determined using a BCA Protein Assay Kit (23225, Thermo Fisher Scientific Inc., Waltham, MA, USA). An equivalent amount of protein (50 μg) was separated using 8% to 12% sodium dodecyl sulfate-polyacrylamide gel electrophoresis and electro-transferred onto a 0.45 μm polyvinylidene fluoride membrane (IPVH0001, Merck KgaA Company, Darmstadt, Germany). The membrane was incubated in 5% (w/v) bovine serum albumin solution prepared in Tris-buffered saline at room temperature for 1 h to block nonspecific binding sites, followed by an overnight incubation with the designated primary antibodies (Table S2) at 4 °C to allow specific antigen-antibody interactions. The membranes were gently washed twice with Tris-buffered saline with Tween 20 (10 min each), followed by 1 h incubation with DyLight 800-labeled secondary antibodies at room temperature. Blots were acquired using the infrared imaging system (Odyssey CLx, LI-COR Biotechnology Company, Lincoln, NE, USA) and subsequently quantified with ImageJ software (National Institutes of Health, Bethesda, MD, USA).

### 2.11 Statistical analysis

Growth performance (BW, ADG, ADFI, and F:G) was subjected to statistical analysis with a pen including 16 pigs as experimental unit. The following parameters, including carcass traits, meat quality, amino acid and fatty acid compositions, serum and SAT biochemical indices, adipocyte areas, as well as mRNA and protein expressions, were subjected to statistical analysis on an individual-pig basis. Before performing the analysis of variance, the underlying assumptions of normality and homogeneity of variance were scrutinized by the application of the Shapiro-Wilk test and Levene’s test, respectively. When necessary, data transformations were applied to ensure compliance with the assumptions of normality and homogeneity of variance. The data among three treatments were subjected to one-way analysis of variance using SAS software (version 9.2, SAS Institute Inc., Cary, NC, USA), with Tukey’s multiple-range tests. The model for the data was the following equation:

$$Y_{ij}=\mu +T_{i}+\varepsilon_{ij}\text{,}$$

where *Y*_*ij*_ is the dependent variable; *μ* is the overall mean; *T*_*i*_ is the fixed effect of treatment; and *ε*_*ij*_ is the random effect. The statistical model incorporated dietary treatment as the sole fixed effect, while pen and individual pig were treated as random effects. For statistical analysis of meat quality, carcass traits, and amino acid composition of LT muscle, the pen-by-individual pig interaction was additionally considered as a random effect.

The data were presented as mean value and standard error of the mean (SEM). Statistical significance was defined as *P* < 0.05, while values within the range of 0.05 ≤ *P* ≤ 0.10 were considered to indicate a trend.

## 3 Results

### 3.1 Growth performance

As shown in Table 3, no differences were observed among dietary treatments in ADG, ADFI, and F:G of finishing pigs (*P* > 0.05).

**Table 3:** Effects of dietary supplementation with *L. reuteri* or *L. johnsonii* on growth in finishing pigs.

Items	Groups^1^			SEM	*P*-value
	CON	*L*. *reuteri*	*L*. *johnsonii*		
Initial BW, kg	110.98	112.58	106.10	2.583	0.235
Final BW, kg	148.44	146.66	142.33	2.419	0.238
ADG, g/d	892	811	862	29.7	0.206
ADFI, g/d	3881	3876	3786	40.9	0.236
F:G	4.39	4.78	4.39	0.129	0.097

*L. reuteri = Lactobacillusreuteri*; *L. johnsonii = Lactobacillus johnsonii;* BW = body weight; ADG = average daily gain; ADFI = average daily feed intake; F:G = feed:gain ratio; SEM = standard error of the mean.

Mean values with different letters are considered significantly different at *P* < 0.05. Data are expressed as the means and SEM (*n* = 6).

1 CON, basal diet; *L. reuteri*, basal diet supplemented with *L. reuteri* at a daily dose of 5 × 10^10^ CFU per pig; *L. johnsonii*, basal diet supplemented with *L. johnsonii* at a daily dose of 5 × 10^10^ CFU per pig.

### 3.2 Carcass traits

As shown in Table 4, *L. johnsonii* supplementation significantly decreased the 10th rib fat thickness (*P* = 0.040) and last rib fat thickness (*P* = 0.025), while significantly increased the fat-free lean index (*P* = 0.015) compared with the CON, but no changes were observed in pigs between *L. johnsonii* and *L. reuteri* supplementations (*P* > 0.05). The hot carcass weight, dressing percentage, loin eye area, shoulder fat thickness, 6th to 7th rib fat thickness, lumbosacral fat thickness and average back-fat depth were not altered by dietary *Lactobacillus* supplementations (*P* > 0.05).

**Table 4:** Effects of dietary supplementation with *L. reuteri* or *L. johnsonii* on carcass traits in finishing pigs.

Items	Groups^1^			SEM	*P*-value
	CON	*L*. *reuteri*	*L*. *johnsonii*		
Carcass weight, kg	113.23	111.91	110.79	2.636	0.809
Dressing percentage, %	76.69	74.90	74.12	1.586	0.512
**Back fat depth, mm**					
Shoulder fat thickness	36.50	35.73	34.09	1.175	0.353
The 6th to 7th rib fat thickness	25.82	22.88	22.24	1.500	0.222
The 10th rib fat thickness	22.49^a^	21.17^ab^	17.59^b^	1.307	0.040
The last rib fat thickness	24.39^a^	20.76^ab^	19.47^b^	1.211	0.025
Lumbosacral fat thickness	11.68	10.24	10.19	0.664	0.222
Average back-fat depth	24.19	22.24	21.25	0.845	0.065
Loin eye area, cm^2^	46.09	49.10	50.32	2.662	0.523
Fat-free lean index	50.88^b^	52.06^ab^	52.43^a^	0.357	0.015

*L. reuteri* = *Lactobacillusreuteri*; *L. johnsonii = Lactobacillusjohnsonii*; SEM = standard error of the mean.

Mean values with different letters are considered significantly different at *P* < 0.05. Data are expressed as the means and SEM (*n* = 8).

1 CON, basal diet; *L. reuteri*, basal diet supplemented with *L. reuteri* at a daily dose of 5 × 10^10^ CFU per pig; *L. johnsonii*, basal diet supplemented with *L. johnsonii* at a daily dose of 5 × 10^10^ CFU per pig.

### 3.3 Meat quality and chemical composition

*L. reuteri* rather than *L. johnsonii* supplementation significantly decreased the shear force and IMF content of LT muscle compared to the CON (*P* < 0.05; Table 5). Nevertheless, there were no changes observed in meat quality, including pH values, flesh color score, L∗, a∗, b∗ values, drip loss, cooking loss, marbling score, and moisture contents, as well as shear force between dietary supplementation with *L. johnsonii* and *L. reuteri* (*P* > 0.05).

**Table 5:** Effects of dietary supplementation with *L. reuteri* or *L. johnsonii* on meat quality in finishing pigs.

Items	Groups^1^			SEM	*P*-value
	CON	*L*. *reuteri*	*L*. *johnsonii*		
**Meat quality**					
pH_45 min_	5.63	5.74	5.65	0.090	0.694
pH_24 h_	5.46	5.48	5.48	0.045	0.893
Flesh color score	3.50	3.56	3.56	0.107	0.894
L∗_45 min_	49.1	47.5	47.9	1.77	0.802
a∗_45 min_	15.2	14.5	14.9	0.29	0.223
b∗_45 min_	3.1	2.9	2.9	0.36	0.896
L∗_24 h_	54.0	51.6	51.5	1.36	0.351
a∗_24 h_	16.2	16.3	15.7	0.45	0.590
b∗_24 h_	7.5	6.9	6.6	0.54	0.519
Drip loss, %	1.62	1.50	1.76	0.218	0.696
Cooking loss, %	19.17	20.68	20.37	1.572	0.775
Shear-force, N	86.99^a^	61.58^b^	63.75^ab^	7.365	0.043
Marbling score	3.75	3.63	3.63	0.286	0.939
Intramuscular fat, %	3.79^a^	2.49^b^	2.70^ab^	0.333	0.026
Moisture, %	72.24	72.60	72.43	0.335	0.759

*L. reuteri* = *Lactobacillusreuteri*; *L. johnsonii = Lactobacillusjohnsonii*; L = lightness; a = redness; b = yellowness; SEM = standard error of the mean.

Mean values with different letters are considered significantly different at *P* < 0.05. Data are expressed as the means and SEM (*n* = 8).

1 CON, basal diet; *L. reuteri*, basal diet supplemented with *L. reuteri* at a daily dose of 5 × 10^10^ CFU per pig; *L. johnsonii*, basal diet supplemented with *L. johnsonii* at a daily dose of 5 × 10^10^ CFU per pig.

### 3.4 Serum biochemical indices

As shown in Table 6, dietary supplementation with *L. johnsonii* rather than *L. reuteri* significantly increased serum IGF-1 concentration compared to the CON (*P* < 0.001). Furthermore, both dietary supplementations with *L. reuteri* and *L. johnsonii* significantly decreased serum cortisol concentration compared to the CON (*P* < 0.001). In addition, the serum concentrations of TG, TC, HDL, LDL, GLU, TP, ALB, and GLB were not altered by dietary treatments (*P* > 0.05).

**Table 6:** Effects of dietary supplementation with *L. reuteri* or *L. johnsonii* on serum biochemical indexes in finishing pigs.

Items	Groups^1^			SEM	*P*-value
	CON	*L*. *reuteri*	*L*. *johnsonii*		
TG, mmol/L	1.37	1.27	1.31	0.061	0.544
TC, mmol/L	2.65	2.49	2.56	0.097	0.532
HDL, mmol/L	0.92	0.77	0.84	0.045	0.107
LDL, mmol/L	1.54	1.46	1.50	0.091	0.858
GLU, g/L	3.49	3.64	3.61	0.164	0.780
TP, g/L	61.15	65.70	63.00	1.337	0.084
ALB, g/L	34.38	34.12	36.18	0.931	0.264
GLB, g/L	26.77	31.58	26.82	1.691	0.101
IGF-1, ng/mL	33.95^b^	30.90^b^	49.52^a^	1.678	<0.001
Cortisol, ng/mL	53.60^a^	37.84^b^	30.36^b^	3.015	<0.001

*L. reuteri* = *Lactobacillusreuteri*; *L. johnsonii = Lactobacillusjohnsonii*; TG = triglyceride; TC = total cholesterol; HDL = high-density lipoprotein; LDL = low-density lipoprotein; GLU = glucose; TP = total protein; ALB = albumin; GLB = globuim; IGF-1 = insulin-like growth factor-1; SEM = standard error of the mean.

Mean values with different letters are considered significantly different at *P* < 0.05. Data are expressed as the means and SEM (*n* = 6).

1 CON, basal diet; *L. reuteri*, basal diet supplemented with *L. reuteri* at a daily dose of 5 × 10^10^ CFU per pig; *L. johnsonii*, basal diet supplemented with *L. johnsonii* at a daily dose of 5 × 10^10^ CFU per pig.

### 3.5 Hydrolytic amino acids and free amino acid profile of the LT muscle

As shown in Table 7, both dietary supplementations with *L. reuteri* and *L. johnsonii* significantly increased free valine concentration in LT muscle compared to the CON (*P* = 0.010). The concentrations of free methionine (*P* = 0.049) and isoleucine (*P* = 0.034) in LT muscle were significantly increased by *L. reuteri* supplementation compared to the CON. In contrast, no changes were observed in these amino acids in pigs that received dietary *L. johnsonii* supplementation (*P* > 0.05). In addition, dietary *L. reuteri* supplementation significantly increased the concentration of free histidine in LT muscle compared to *L. johnsonii* supplementation (*P* = 0.048), but no changes were observed in pigs between *L. johnsonii* supplementation and the CON (*P* > 0.05). The concentrations of both hydrolytic essential amino acids and non-essential amino acids in LT muscle were not influenced by the dietary *Lactobacillus* supplementations (*P* > 0.05; Table 8).

**Table 7:** Effects of dietary supplementation with *L. reuteri* or *L. johnsonii* on free amino acids concentrations of the longissimus thoracis (LT) muscle in finishing pigs (mg/100 g muscle based on wet weight).

Items	Groups^1^			SEM	*P*-value
	CON	*L*. *reuteri*	*L*. *johnsonii*		
**Essential amino acids**					
Lysine	3.01	3.19	3.23	0.223	0.761
Methionine	2.05^b^	2.69^a^	2.53^ab^	0.179	0.049
Tryptophan	2.55	2.87	2.63	0.157	0.346
Threonine	2.93	3.23	3.06	0.213	0.608
Valine	3.97^b^	5.52^a^	5.61^a^	0.384	0.010
Isoleucine	2.26^b^	3.01^a^	2.75^ab^	0.191	0.034
Leucine	4.00	4.73	4.61	0.270	0.150
Arginine	0.64	0.76	0.73	0.065	0.442
Histidine	4.17^ab^	4.53^a^	3.57^b^	0.261	0.048
Phenylalanine	4.99	5.86	5.71	0.316	0.139
**Non-essential amino acids**					
Alanine	3.63	3.40	4.01	0.241	0.219
Aspartic acid	0.62	0.52	0.72	0.104	0.439
Glutamic acid	2.52	2.41	2.46	0.200	0.927
Glycine	4.10	4.30	4.27	0.192	0.743
Proline	1.40	1.41	1.46	0.086	0.877
Serine	1.93	2.14	2.06	0.132	0.543
Tyrosine	3.27	4.09	3.89	0.335	0.221

*L. reuteri* = *Lactobacillusreuteri*; *L. johnsonii = Lactobacillusjohnsonii*; SEM = standard error of the mean.

Mean values with different letters are considered significantly different at *P* < 0.05. Data are expressed as the means and SEM (*n* = 8).

1 CON, basal diet; *L. reuteri*, basal diet supplemented with *L. reuteri* at a daily dose of 5 × 10^10^ CFU per pig; *L. johnsonii*, basal diet supplemented with *L. johnsonii* at a daily dose of 5 × 10^10^ CFU per pig.

**Table 8:** Effects of dietary supplementation with *L. reuteri* or *L. johnsonii* on hydrolytic amino acid concentrations of the longissimus thoracis (LT) muscle in finishing pigs (g/100 g muscle based on wet weight).

Items	Groups^1^			SEM	*P*-value
	CON	*L*. *reuteri*	*L*. *johnsonii*		
**Essential amino acids**					
Lysine	1.92	2.02	1.97	0.040	0.266
Methionine	0.61	0.64	0.61	0.013	0.252
Tryptophan	0.28	0.27	0.26	0.011	0.483
Threonine	0.96	1.00	0.98	0.020	0.306
Valine	1.09	1.14	1.12	0.021	0.246
Isoleucine	1.02	1.07	1.04	0.020	0.227
Leucine	1.71	1.79	1.75	0.035	0.279
Arginine	1.34	1.40	1.37	0.028	0.301
Histidine	1.00	1.05	0.99	0.025	0.255
Phenylalanine	0.83	0.88	0.85	0.016	0.234
**Non-essential amino acids**					
Alanine	1.20	1.26	1.23	0.024	0.299
Aspartic acid	1.97	2.06	2.01	0.040	0.296
Glutamic acid	2.97	3.12	3.04	0.064	0.251
Glycine	0.91	0.94	0.92	0.018	0.446
Proline	0.78	0.82	0.80	0.016	0.249
Serine	0.80	0.83	0.81	0.016	0.330
Tyrosine	0.71	0.75	0.72	0.014	0.219

*L. reuteri* = *Lactobacillusreuteri*; *L. johnsonii = Lactobacillusjohnsonii*; SEM = standard error of the mean.

Mean values with different letters are considered significantly different at *P* < 0.05. Data are expressed as the means and SEM (*n* = 8).

1 CON, basal diet; *L. reuteri*, basal diet supplemented with *L. reuteri* at a daily dose of 5 × 10^10^ CFU per pig; *L. johnsonii*, basal diet supplemented with *L. johnsonii* at a daily dose of 5 × 10^10^ CFU per pig.

### 3.6 Fatty acid composition of the LT muscle

Compared to the CON, both *L. reuteri* and *L. johnsonii* supplementation significantly decreased the concentrations of C16:0, C18:0 and saturated fatty acids (SFA) (*P* < 0.05; Table 9). Moreover, the C21:0 concentration was decreased by *L. reuteri* supplementation relative to the CON (*P* = 0.034), but was not altered by *L. johnsonii* supplementation (*P* > 0.05). In addition, the concentrations of monounsaturated fatty acids (MUFA), polyunsaturated fatty acids (PUFA), n-3 PUFA and n-6 PUFA in LT muscle of finishing pigs were not altered by dietary *Lactobacillus* supplementation (*P* > 0.05).

**Table 9:** Effects of dietary supplementation with *L. reuteri* or *L. johnsonii* on fatty acid concentrations in the longissimus thoracis (LT) muscle of finishing pigs.

Items	Groups^1^			SEM	*P*-value
	CON	*L*. *reuteri*	*L*. *johnsonii*		
**Fatty acids concentrations, mg/100 g muscle based on wet weight**					
C8:0	0.68	0.51	0.56	0.083	0.369
C10:0	3.65	2.59	3.16	0.625	0.500
C12:0	2.90	2.04	2.16	0.592	0.548
C14:0	58.48	30.56	31.34	8.570	0.059
C14:1	0.79	0.53	0.64	0.185	0.628
C15:0	0.97	0.74	0.76	0.187	0.633
C16:0	1086.41^a^	586.00^b^	606.77^b^	127.479	0.023
C16:1	87.80	65.66	77.94	19.836	0.736
C17:0	5.23	3.75	3.78	1.012	0.515
C18:0	590.78^a^	330.99^b^	330.77^b^	66.020	0.020
C18:1n9c	1226.23	817.46	941.07	253.357	0.519
C18:2n6c	205.42	234.54	239.43	28.358	0.664
C18:3n3	9.89	6.61	6.73	2.080	0.468
C20:0	8.25	5.87	6.41	1.677	0.587
C20:1	23.45	16.98	17.84	4.953	0.614
C21:0	14.98^a^	8.32^b^	9.21^ab^	1.748	0.034
C20:3n6	5.60	5.12	5.91	0.662	0.704
C20:4n6	39.86	39.95	44.50	4.234	0.683
C20:3n3	3.33	2.97	2.63	1.006	0.887
C22:0	1.33	1.26	1.36	0.178	0.921
C20:5n3	1.33	1.24	1.46	0.151	0.578
C22:1n9	5.15	4.54	5.85	0.656	0.392
C23:0	0.75	0.67	0.68	0.109	0.874
C24:0	1.75	1.40	1.89	0.244	0.358
C24:1	1.87	1.86	2.10	0.197	0.630
C22:6n3	1.80	1.64	1.85	0.238	0.818
SFA	1776.14^a^	974.67^b^	998.86^b^	190.146	0.014
MUFA	1345.28	907.02	1045.45	274.081	0.527
PUFA	267.22	292.08	302.50	28.660	0.677
n-3 PUFA	16.34	12.47	12.66	3.291	0.652
n-6 PUFA	250.88	279.61	289.83	28.378	0.613
n-6/n-3 PUFA	2360.37	2808.63	2408.35	573.235	0.834
PUFA/MUFA	28.87	41.66	32.92	7.798	0.511
UFA/SFA	95.41	139.55	137.61	25.213	0.399

*L. reuteri* = *Lactobacillusreuteri*; *L. johnsonii = Lactobacillusjohnsonii*; SFA = saturated fatty acid; MUFA = monounsaturated fatty acids; PUFA = polyunsaturated fatty acids; SEM = standard error of the mean.

Mean values with different letters are considered significantly different at *P* < 0.05. Data are expressed as the means and SEM (*n* = 6).

1 CON, basal diet; *L. reuteri*, basal diet supplemented with *L. reuteri* at a daily dose of 5 × 10^10^ CFU per pig; *L. johnsonii*, basal diet supplemented with *L. johnsonii* at a daily dose of 5 × 10^10^ CFU per pig.

### 3.7 Serum inflammatory factors and antioxidant capacity

Serum inflammatory factors and key indicators of antioxidant capacity were also evaluated (Table 10). Dietary *L.* *johnsonii* supplementation, but not *L. reuteri*, significantly decreased serum LPS concentration in finishing pigs compared to the CON (*P* = 0.005). Compared to the CON, both *L. reuteri* and *L. johnsonii* supplementation significantly decreased serum concentrations of TNF-α (*P* = 0.002), IL-1β (*P* < 0.001), and IL-17 (*P* = 0.001). Moreover, both *L. reuteri* and *L. johnsonii* supplementation increased IL-4 level compared to the CON (*P* < 0.001), and *L. johnsonii* was more effective than *L. reuteri* in elevating IL-4 level. Dietary supplementation with *L. johnsonii* rather than *L. reuteri* significantly increased serum IL-10 concentration compared to the CON (*P* = 0.001). Furthermore, both dietary supplementation with *L. reuteri* and *L. johnsonii* significantly increased T-AOC in serum (*P* = 0.004). Dietary *L.* *johnsonii* supplementation significantly increased serum SOD activity compared to the CON (*P* = 0.004). Notably, dietary *L. reuteri* supplementation significantly decreased CAT activity compared to the CON (*P* = 0.037). *L. reuteri* supplementation significantly decreased serum GSH-Px activity compared to *L. johnsonii* supplementation and the CON (*P* < 0.001). Serum IL-6 and MDA levels were not altered by dietary *Lactobacillus* supplementations (*P* > 0.05).

**Table 10:** Effects of dietary supplementation with *L. reuteri* or *L. johnsonii* on serum inflammatory factors and antioxidant capacity in finishing pigs.

Items	Groups^1^			SEM	*P*-value
	CON	*L*. *reuteri*	*L*. *johnsonii*		
**Inflammatory factors**					
LPS, pg/mL	226.88^a^	218.74^a^	167.84^b^	11.419	0.005
TNF-α, ng/mL	1.78^a^	1.33^b^	1.12^b^	0.107	0.002
IL-1β, pg/mL	148.55^a^	89.59^b^	74.35^b^	8.202	<0.001
IL-6, ng/mL	14.28	12.03	12.10	1.054	0.262
IL-17, ng/mL	16.94^a^	11.08^b^	9.20^b^	1.201	0.001
IL-4, pg/mL	1016.90^c^	1359.85^b^	1590.44^a^	50.476	<0.001
IL-10, pg/mL	268.64^b^	302.97^b^	343.85^a^	11.025	0.001
**Antioxidant capacity**					
T-AOC, U/mL	13.31^b^	17.40^a^	19.01^a^	1.013	0.004
SOD, U/mL	125.66^b^	183.65^ab^	216.54^a^	16.274	0.004
CAT, U/mL	341.04^a^	262.99^b^	279.03^ab^	20.220	0.037
GSH-Px, U/mL	2.58^a^	1.95^b^	2.27^a^	0.084	<0.001
MDA, nmol/mL	0.28	0.31	0.31	0.032	0.753

*L. reuteri* = *Lactobacillusreuteri*; *L. johnsonii = Lactobacillusjohnsonii*; LPS = lipopolysaccharide; TNF-α = tumor necrosis factor-α; Il-1β = interleukin-1β; IL-6 = interleukin-6; IL-17 = interleukin-17; IL-4 = interleukin-4; IL-10 = interleukin-10; T-AOC = total antioxidant capacity; SOD = superoxide dismutase; CAT = catalase; GSH-Px = glutathione peroxidase; MDA = malondialdehyde; SEM = standard error of the mean.

Mean values with different letters are considered significantly different at *P* < 0.05. Data are expressed as the means and SEM (*n* = 6).

1 CON, basal diet; *L. reuteri*, basal diet supplemented with *L. reuteri* at a daily dose of 5 × 10^10^ CFU per pig; *L. johnsonii*, basal diet supplemented with *L. johnsonii* at a daily dose of 5 × 10^10^ CFU per pig.

### 3.8 Morphological structure of subcutaneous adipose tissue

**Fig. 1:**
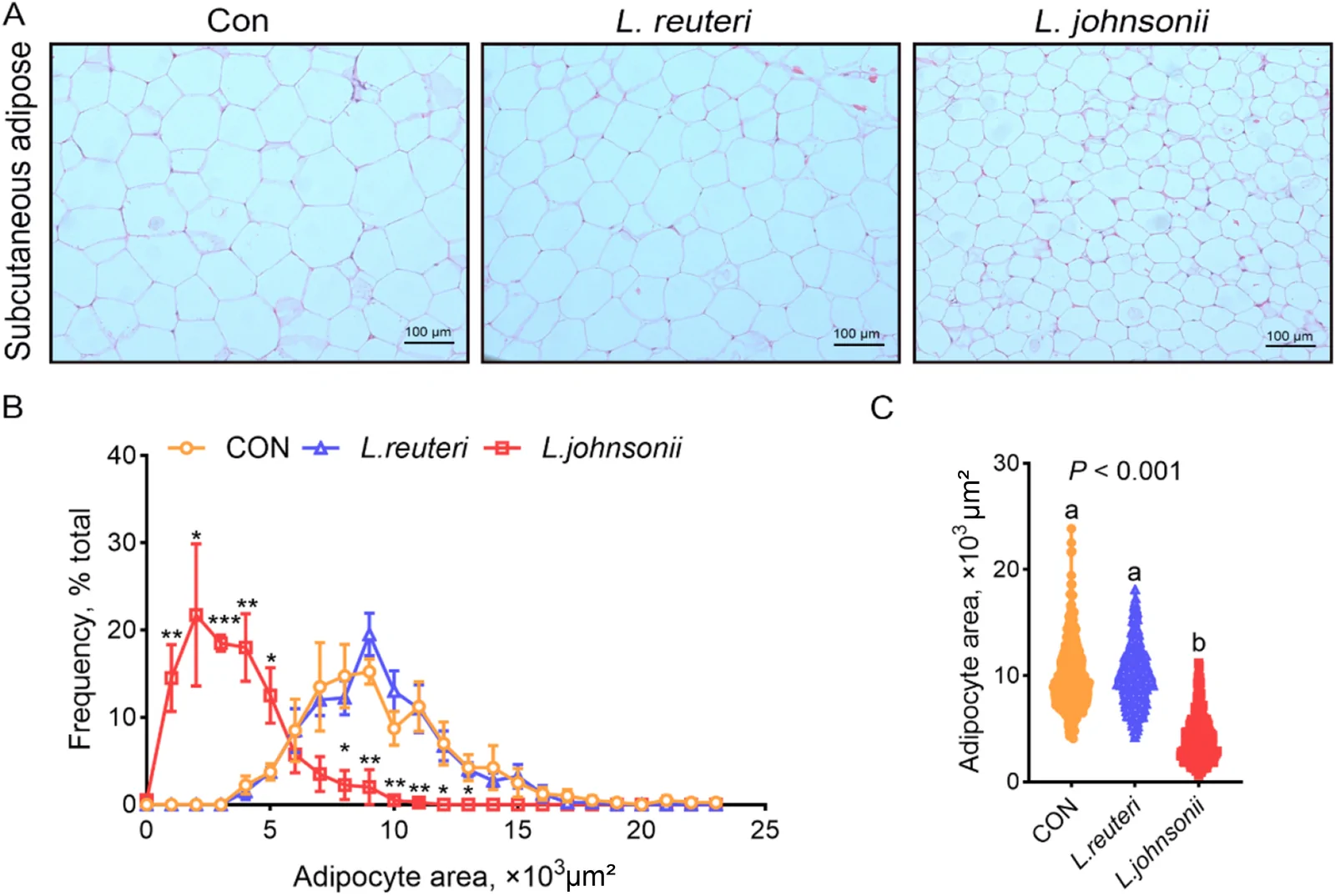
Morphological structure of subcutaneous adipose tissue. Hematoxylin and eosin (H&E) staining (A) and adipocyte area (B and C) in the subcutaneous adipose tissue of finishing pigs. Scale bar, 100 μm. CON, basal diet; *L. reuteri*, basal diet supplemented with *L. reuteri* at a daily dose of 5 × 10^10^ CFU per pig; *L. johnsonii*, basal diet supplemented with *L. johnsonii* at a daily dose of 5 × 10^10^ CFU per pig. Statistics are performed with one-way ANOVA, followed by Tukey’s multiple-range test. Data are expressed as the means and SEM (*n* = 4). Different letters are considered significantly different at *P* < 0.05. ∗ *P* < 0.05, ∗∗ *P* < 0.01, ∗∗∗ *P* < 0.001. *L. reuteri* = *Lactobacillusreuteri*; *L. johnsonii = Lactobacillusjohnsonii*; SEM = standard error of the mean.

As shown in Fig. 1A–C, results of H&E staining analysis demonstrated that dietary supplementation with *L. johnsonii* significantly decreased the adipocyte area in SAT compared to *L. reuteri* supplementation and the CON (*P* < 0.001), but no change was observed between *L. reuteri* supplementation and the CON (*P* > 0.05).

### 3.9 mRNA expressions of genes related to lipid metabolism in subcutaneous adipose tissue

**Fig. 2:**
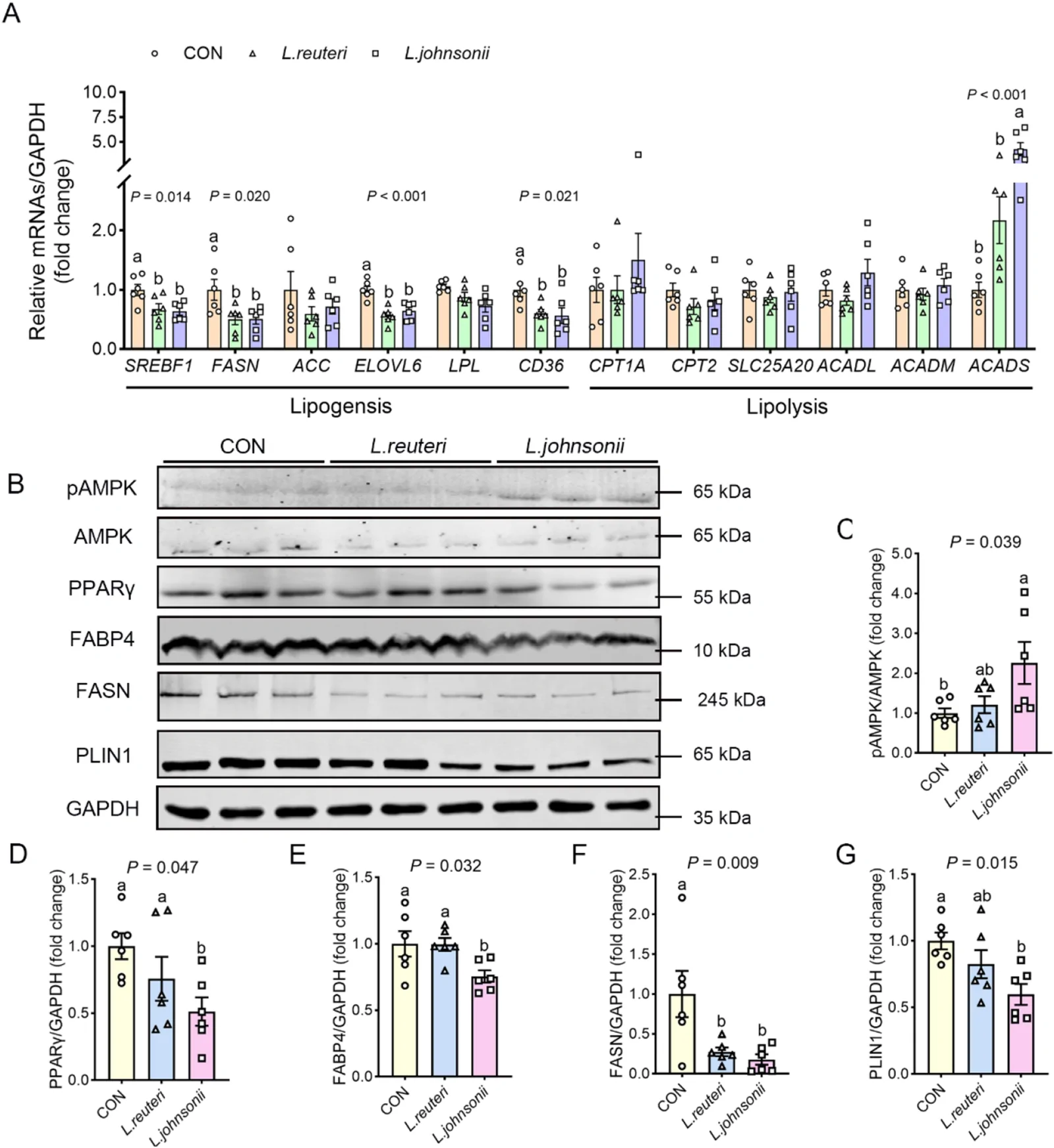
Effects of dietary supplementation with *L. reuteri* or *L. johnsonii* on lipid metabolism in the subcutaneous adipose tissue (SAT) of finishing pigs. (A) The relative mRNA expression levels of lipogenesis and lipolysis genes in the SAT of finishing pigs. (B–G) Western blotting analysis and quantification of pAMPK, AMPK, PPARγ, FABP4, FASN and PLIN1 in the SAT of finishing pigs. CON, basal diet; *L. reuteri*, basal diet supplemented with *L. reuteri* at a daily dose of 5 × 10^10^ CFU per pig; *L. johnsonii*, basal diet supplemented with *L. johnsonii* at a daily dose of 5 × 10^10^ CFU per pig. Statistics are performed with one-way ANOVA, followed by Tukey’s multiple-range test. Data are expressed as the means and SEM (*n* = 6). Different letters are considered significantly different at *P* < 0.05. *L. reuteri* = *Lactobacillusreuteri*; *L. johnsonii = Lactobacillusjohnsonii*; SEM = standard error of the mean.

Given that dietary supplementation with *L. johnsonii* decreased backfat thickness in finishing pigs, as reflected by smaller adipocytes in SAT, the mRNA expression of lipid metabolism-related genes in SAT was further analyzed (Fig. 2A). Both dietary supplementation with *L. johnsonii* and *L. reuteri* significantly decreased mRNA expressions of *SREBF1*, *FASN*, *ELOVL6*, and *CD36* compared to the CON (*P* < 0.05). Dietary supplementation with *L. johnsonii* rather than *L. reuteri* significantly increased *ACADS* mRNA expression compared to the CON (*P* < 0.001).

In addition, the mRNA expression levels of *ACC*, *LPL*, *CPT1A*, *CPT2*, *SLC25A20*, *ACADL* and *ACADM* were not altered by dietary *Lactobacillus* supplementations (*P* > 0.05).

### 3.10 Enzyme activities and protein expression levels of lipid metabolism in subcutaneous adipose tissue

Dietary supplementation with *L. johnsonii* rather than *L. reuteri* significantly increased ATGL activity in SAT compared to the CON (*P* = 0.019; Table 11). In contrast, *L. reuteri r*ather than *L. johnsonii* supplementation significantly increased HSL activity relative to the CON (*P* = 0.032). Consistent to the results on mRNA expression levels, *L. johnsonii* supplementation, instead of *L. reuteri* supplementation, significantly decreased SCD1 activity relative to the CON (*P* = 0.012). The ACC activity was not affected by dietary *Lactobacillus* supplementations (*P* > 0.05).

**Table 11:** Effects of dietary supplementation with *L. reuteri* or *L. johnsonii* on enzyme activities of lipid metabolism in subcutaneous adipose tissue of finishing pigs.

Items	Groups^1^			SEM	*P*-value
	CON	*L*. *reuteri*	*L*. *johnsonii*		
ATGL, U/mg prot	112.64^b^	185.12^ab^	228.92^a^	25.712	0.019
HSL, mU/mg prot	3.24^b^	4.77^a^	3.53^ab^	0.409	0.032
SCD1, U/mg prot	0.38^a^	0.38^a^	0.21^b^	0.040	0.012
ACC, mU/mg prot	25.35	38.62	32.89	3.984	0.093

*L. reuteri* = *Lactobacillusreuteri*; *L. johnsonii = Lactobacillusjohnsonii*; ATGL = adipose triglyceride lipase; HSL = hormone-sensitive lipase; SCD1 = stearoyl-CoA desaturase 1; ACC = acetyl-CoA carboxylase; SEM = standard error of the mean.

Mean values with different letters are considered significantly different at *P* < 0.05. Data are expressed as the means and SEM (*n* = 6).

1 CON, basal diet; *L. reuteri*, basal diet supplemented with *L. reuteri* at a daily dose of 5 × 10^10^ CFU per pig; *L. johnsonii*, basal diet supplemented with *L. johnsonii* at a daily dose of 5 × 10^10^ CFU per pig.

Western blot analysis further revealed that dietary *L. johnsonii* rather than *L. reuteri* supplementation significantly increased protein level of *pAMPK*/*AMPK* (*P* = 0.039; Fig. 2C), while decreasing protein levels of PPARγ, FABP4, and PLIN1 (*P* < 0.05; Fig. 2D, E and G) compared to the CON. Compared to the CON, both dietary supplementation with *L. reuteri* and *L. johnsonii* significantly decreased FASN protein level (*P* = 0.009; Fig. 2F).

## 4 Discussion

Recently, the application of *Lactobacilli* as a probiotic in the pig industry has become increasingly prominent (Patil et al., 2020). Studies have shown that *Lactobacilli* not only promote growth and prevent diarrhea in piglets, but also alleviate stress responses and enhance overall health by maintaining intestinal microbiota homeostasis and supporting normal immune function (Hou et al., 2015). The present study demonstrated that dietary *L. reuteri* or *L. johnsonii* supplementation did not affect the growth performance of heavy finishing pigs (approximately during 110–150 kg BW), which was consistent with the previous research (Tian et al., 2021). Accordingly, the growth-promoting efficacy of *Lactobacillus* was inferred to be pronounced in neonatal and weaned pigs rather than finishing pigs.

Studies of *Lactobacilli* supplementation on lipid metabolism mainly focused on mouse models. It has been shown that gastric infusion with *Lactobacilli* *plantarum* HF02 reduced adipocyte volume in epididymal fat by 25.45% in high-fat diet (HFD)-induced obese mice (Chen et al., 2023). Similarly, intervention with *L. paracasei* reversed HFD-induced adipocyte hypertrophy in mice (Ji et al., 2022). Research on finishing pigs remains severely inadequate, especially in heavy-weight pigs. This study showed that, instead of *L. reuteri*, dietary *L. johnsonii* supplementation could significantly reduce body fat deposition in pigs compared to the CON pigs, demonstrated by the reduced size of SAT adipocytes and subcutaneous backfat thickness. In particular, dietary *L. johnsonii* supplementation significantly decreased backfat thickness at the 10th rib by 21.79% and last rib by 20.17% in finishing pigs, while significantly increasing the fat-free lean index without compromising growth performance and IMF content. It suggested that *L. johnsonii* may enhance carcass quality through its specific modulation of fat deposition.

Body fat deposition is governed by the metabolic equilibrium between lipogenesis and lipolysis, and its core regulatory mechanism involves a cascade of the AMPK signaling pathway and adipogenic transcription factors. As an “intracellular energy sensor”, AMPK serves as a pivotal regulator that orchestrates fatty acid synthesis and oxidation, cholesterol metabolism, etc. by monitoring cellular energy status (Göransson et al., 2023). Those findings demonstrated that dietary *L. johnsonii* supplementation significantly increased the phosphorylation level of AMPK in SAT of finishing pigs, suggesting that *L. johnsonii* may play a regulatory role through activation of the AMPK signaling pathway. In the regulation of adipocyte differentiation and lipid metabolism, CCAAT/enhancer-binding protein (CEBP) β and CEBPδ initiate the adipogenic differentiation of fibro-adipogenic progenitors, and then CEBPα and PPARγ are activated. They promoted lipid accumulation by up-regulating the expression of downstream target genes involved in fatty acid uptake and synthesis (Lefterova and Lazar, 2009). Here, the results revealed that dietary *L. johnsonii* supplementation significantly reduced the expression level of *PPARγ* in SAT of finishing pigs, indicating that *L. johnsonii* may regulate the expression of PPARγ through activating the AMPK-mediated pathway, thereby affecting the lipid metabolism process.

Similarly, *Lactobacilli* *fermentum* MG4231 and MG4244 had been shown to enhance the phosphorylation activity of HSL through the AMPK signaling pathway in adipogenesis of 3T3-L1 pre-adipocytes, thereby inhibiting lipid accumulation (Kim et al., 2020). As key rate-limiting enzymes in lipolysis, both HSL and ATGL are regulated by AMPK. Specifically, ATGL primarily catalyzes the hydrolysis of triglycerides to generate diacylglycerols, while HSL functions as a diacylglycerol lipase (Jang et al., 2025). In the present study, a down-regulation of the expression of lipid synthesis molecules (*SREBF1*, *FASN*, *ELOVL6*, *CD36*, FABP4, and PLIN1) and an up-regulation of the expression of lipid catabolism-related molecules (ATGL and *ACADS*) in SAT of finishing pigs. These findings suggested that *L. johnsonii* can effectively reduce SAT deposition in finishing pigs by activating the AMPK/PPARγ signaling pathway, which enhances lipolysis and depresses lipid synthesis.

Furthermore, *L. johnsonii* supplementation significantly increased serum IGF-1 levels in finishing pigs. Considering the function of IGF-1 promoting muscle protein synthesis (Huang et al., 2006), *L. johnsonii* might promote protein deposition in muscle tissues and ultimately increase the fat-free lean index, which warrants further validation. Shear force exhibited a strong negative correlation with pork tenderness, whereby decreased shear force values represented improved tenderness and a softer meat texture (Yan et al., 2023). In this study, dietary supplementation with *L. reuteri* rather than *L. johnsonii* in finishing pigs significantly reduced LT muscle shear force, which was consistent with previous studies in piglets (Tian et al., 2021). This study confirmed that *L. reuteri* could also improve pork tenderness in heavy finishing pigs.

In this study, it was not observing that *L. reuteri* supplementation reduced LT muscle drip loss, which may be due to the considerably low level of drip loss observed in the CON. The higher pork shear force in the CON pigs might be attributed to the substantially heavier carcass weight (exceeding 110 kg). In addition, it was found that *L. reuteri* significantly increased the levels of essential amino acids in fresh meat, including free methionine, valine, isoleucine, and histidine.

Notably, both dietary supplementation with *L. reuteri* and *L. johnsonii* significantly reduced SFA concentration in LT muscle of finishing pigs, which is beneficial to consumer health because SFA intake contributes to cardiovascular disease risk (Dicks, 2024; Siri-Tarino et al., 2015). Moreover, relative to the CON pigs, the variations in IMF content in LT muscle of pigs received *L. reuteri* and *L. johnsonii* did not parallel the changes in backfat thickness. Dietary *L.* *reuteri* supplementation, instead of *L. johnsonii*, significantly reduced IMF content in finishing pigs compared to CON pigs. Although the dynamic changes in IMF content are known to be not entirely consistent with those of backfat deposition (Zhou et al., 2007), the results require further validation through an expanded sample size.

Collectively, above results suggested that dietary supplementation with *L. johnsonii* and *L. reuteri* might exert different roles in fat mobilization, deposition and distribution.

Serum cortisol concentration reflects stress level of animals (Ortiz et al., 2022). *Lactobacilli* could improve immunity and reduce diarrhea in piglets by reducing serum cortisol and TNF-α levels (Kang et al., 2021). However, research on the effect of *Lactobacilli* on stress in finishing pigs is very limited. Results demonstrated that dietary supplementation of either *L. reuteri* or *L. johnsonii* significantly reduced serum cortisol levels, indicating that both *Lactobacillus* strains could effectively relieve the stress in finishing pigs. *Lactobacilli* can alleviate oxidative stress by inducing an increase in the activity of antioxidant enzymes, e.g., GSH-Px, SOD, and CAT (Wang et al., 2009; Zhao et al., 2023). Consistently, in this study, dietary supplementations with either *L. reuteri* or *L. johnsonii* significantly elevated serum T-AOC, indicating both *Lactobacillus* strains effectively enhanced the antioxidant capacity of heavy finishing pigs. Considering the enzymatic and non-enzymatic antioxidant activities of *L. reuteri* fermentation products, the elevation of T-AOC alongside decreased CAT and GSH-Px activities is not contradictory. This suggested that *L. reuteri* supplementation alleviated oxidative stress and increased T-AOC in heavy finishing pigs, while specifically reducing the activities of CAT and GSH-Px (Sies et al., 2017).

Inflammatory processes can induce oxidative stress, while oxidative stress, in turn, aggravates the inflammatory response through the activation of multiple signaling pathways (García-Giménez et al., 2025; Wang et al., 2023a). In this study, *L. reuteri* and *L. johnsonii* showed comparable efficacy in attenuating inflammatory responses, as both significantly reduced the levels of pro-inflammatory cytokines TNF-α, IL1β, and IL-17, while increasing the expression of anti-inflammatory cytokines IL-4, which might be attributed to the beneficial effect of *Lactobacilli* colonization in the gut (Minj et al., 2021). Elevated levels of LPS not only activated the expression of pro-inflammatory cytokines, but also impaired intestinal barrier function and triggered systemic oxidative stress (Weng et al., 2025; Xie et al., 2025). Oxidative stress and inflammatory responses disrupt signaling pathways modulating lipid metabolism, thereby suppressing lipolysis and promoting fat deposition (Furukawa et al., 2004). In this study, *L. johnsonii* significantly reduced serum LPS content in finishing pigs, suggesting that dietary supplementation with *L. johnsonii* might be beneficial to intestinal integrity.

Notably, both *L. reuteri* and *L. johnsonii* effectively alleviated stress and inflammatory responses in finishing pigs, however, only *L. johnsonii* demonstrated a significant reduction in backfat thickness, as proved by the activation of the AMPK pathway by *L. johnsonii* supplementation in SAT of finishing pigs. The difference implied that the two *Lactobacilli* exerted distinct roles in cascade mechanisms of regulating fat accumulation.

## 5 Conclusions

In summary, dietary supplementation with *L. reuteri* and *L. johnsonii* exhibited distinct effects in finishing pigs, even though both of them enhanced antioxidant capacity and reduced stress and inflammatory responses. Dietary *L.* *johnsonii* supplementation promoted lipid catabolism and reduced subcutaneous fat deposition via activating the AMPK/PPARγ signaling pathway, effectively improving carcass quality, while *L. reuteri* supplementation mainly improved meat tenderness. These findings might offer novel insights into the application of *Lactobacillus* supplementation in heavy finishing pigs.

## CRediT authorship contribution statement

**Boyang Wan:** Writing – original draft, Visualization, Resources, Methodology, Investigation, Formal analysis. **Qiuyu Zeng:** Resources, Methodology, Investigation. **Linjuan He:** Resources, Methodology, Investigation. **Xu Qin:** Resources, Investigation. **Mingyang Tan:** Resources, Investigation. **Enfa Yan:** Resources, Investigation. **Jialong Liao:** Resources, Investigation. **Zhongchao Li:** Investigation. **Xin Zhang:** Methodology, Investigation, Funding acquisition. **Jingdong Yin:** Writing – review & editing, Supervision, Investigation, Funding acquisition, Data curation, Conceptualization.

## Declaration of competing interest

The authors declare the following financial interests/personal relationships which may be considered as potential competing interests: Zhongchao Li is currently employed by Guizhou Fuzhiyuan Company (Guiyang, Guizhou, China).
