# Supplementary material for: Distinct functional roles of Lactobacillus strains in modulating lipid metabolism, adipose accumulation, inflammatory responses, and carcass characteristics in heavy finishing pigs
Source: Anim Nutr. 2026 Jul 31;27:143–54. doi: 10.1016/j.aninu.2026.03.006 (PMC13572313; doi:10.1016/j.aninu.2026.03.006)
Supplement: Supplementary file 1 [file mmc1.docx]

Abbreviations and full names of genes and proteins.

| Abbreviations | full names |
| --- | --- |
| **Genes** |  |
| *ACADL* | Acyl-CoA dehydrogenase long chain |
| *ACADM* | Acyl-CoA dehydrogenase medium chain |
| *ACADS* | Acyl-CoA dehydrogenase short chain |
| *ACC* | Acetyl-CoA carboxylase alpha |
| *CD36* | Cluster of differentiation 36 |
| *CPT1A* | Carnitine palmitoyltransferase 1A |
| *CPT2* | Carnitine palmitoyltransferase 2 |
| *ELOVL6* | Elongation of very long chain fatty acids protein 6 |
| *FASN* | Fatty acid synthase |
| *LPL* | Lipoprotein lipase |
| *SLC25A20* | Solute carrier family 25 member 20 |
| *SREBF1* | Sterol regulatory element binding transcription factor 1 |
| **Proteins** |  |
| AMPK | AMP-activated protein kinase |
| FABP4 | Fatty acid binding protein 4 |
| FASN | Fatty acid synthase |
| GAPDH | Glyceraldehyde-3-phosphate dehydrogenase |
| PLIN1 | Perilipin 1 |
| PPARγ | Peroxisome proliferator-activated receptor gamma |

**Table S1** Primer sequences used for quantitative real-time PCR.

| Genes | Primer sequence (5'-3') | Size, bp | Accession No. |
| --- | --- | --- | --- |
| *SREBF1* | F: AAGCGGACGGCTCACAA  R:GCAAGACGGCGGATTTATT | 121 | NM_214157.1 |
| *FASN* | F: GCCTAACTCCTCGCTGCAAT  R: TCCTTGGAACCGTCTGTGTTC | 195 | NM_001099930.1 |
| *ACC* | F: AAATCCACAATGCCAACCC  R: TCACTGTTCCTTCCACCTCC | 87 | XM_021066234.1 |
| *ELOVL6* | F: AGCAGTTCAACGAGAACGAAGCC  R: TGCCGACCGCCAAAGATAAAG | 103 | XM_021100706.1 |
| *LPL* | F: CTCGTGCTCAGATGCCCTAC  R: GGCAGGGTGAAAGGGATGTT | 148 | NM_214286.1 |
| *CD36* | F: TAGGAATCCCACTGCCTCACT  R: GCCCCACCTCCAAGTATCCA | 72 | NM_001044622.1 |
| *CPT1A* | F: ATTACGACGGCAGGCTGTTGAAG  R: AATAGGCTTGGCGACACTTGGC | 153 | NM_001129805.2 |
| *CPT2* | F: CTGACTGCTCGAAACCCCAT  R: GAACACTTCCGGCTCCAAGA | 168 | NM_001246243.1 |
| *SLC25A20* | F: GATGATCCGAGCCTTCCCAG  R: CAGACTTCGGCCTCACAAGT | 103 | XM_003483178.4 |
| *ACADL* | F: CGCTGCTTCCATGGCAAAAT  R: TCGGGCATCCACATAAGCTC | 130 | NM_213897.1 |
| *ACADM* | F: GAACTGGCGAGTACCCTGTC  R: GGCACTTGCCCCAGAGTATT | 190 | NM_214039.1 |
| *ACADS* | F: TTTGAGGACTGTCGCATCCC  R: TTGCCGGCTCCTTGATGAAT | 316 | NM_213898.1 |
| *GAPDH* | F: TCGGAGTGAACGGATTTGGC  R: TGACAAGCTTCCCGTTCTCC | 189 | NM_001206359.1 |

**Table S2** Primary antibodies used for western blot.

| Antibody | Identifier | Source | Country | Dilution |
| --- | --- | --- | --- | --- |
| Anti-pAMPK (Thr172) | 2535 | Cell Signaing technology, Inc. | Danvers, MA, USA | 1:1000 |
| Anti-AMPK | 2532 | Cell Signaing technology, Inc. | Danvers, MA, USA | 1:1000 |
| Anti-PPARγ | 95,128 | Cell Signaing technology, Inc. | Danvers, MA, USA | 1:1000 |
| Anti-FABP4 | ab92501 | abcam Trading (Shanghai) Co., Ltd. | Shanghai, China | 1:1000 |
| Anti-FASN | 66591-1-Ig | Proteintech Group, Inc. | Rosemont, IL, USA | 1:1000 |
| Anti-PLIN1 | 9349 | Cell Signaing technology, Inc. | Danvers, MA, USA | 1:1000 |
| Anti-GAPDH | 2118 | Cell Signaing technology, Inc. | Danvers, MA, USA | 1:10,000 |
| Anti-rabbit IgG (H + L) | 5151 | Cell Signaling Technology, Inc. | Danvers, MA, USA | 1:10,000 |
| Anti-mouse IgG (H + L) | 5257 | Cell Signaling Technology, Inc. | Danvers, MA, USA | 1:10,000 |

IgG = immunoglobulin G.
